# Focal Adhesion-Related Signatures Predict the Treatment Efficacy of Chemotherapy and Prognosis in Patients with Gastric Cancer

**DOI:** 10.3389/fonc.2022.808817

**Published:** 2022-05-04

**Authors:** Xiaohuan Tang, Xiaolong Wu, Ting Guo, Fangzhou Jia, Ying Hu, Xiaofang Xing, Xiangyu Gao, Ziyu Li

**Affiliations:** ^1^ Key Laboratory of Carcinogenesis and Translational Research (Ministry of Education), Department of Gastrointestinal Cancer Center, Peking University Cancer Hospital & Institute, Beijing, China; ^2^ Biological Sample Bank, Peking University Cancer Hospital & Institute, Beijing, China

**Keywords:** gastric cancer, focal adhesion, treatment efficacy, prognosis, tumor microenvironment

## Abstract

**Background:**

The current tumor-node-metastasis (TNM) staging system is insufficient for predicting the efficacy of chemotherapy in patients with gastric cancer (GC). This study aimed to analyze the association between the focal adhesion pathway and therapeutic efficacy of chemotherapy in patients with GC.

**Methods:**

RNA sequencing was performed on 33 clinical samples from patients who responded or did not respond to treatment prior to neoadjuvant chemotherapy. The validation sets containing 696 GC patients with RNA data from three cohorts (PKUCH, TCGA, and GSE14210) were analyzed. A series of machine learning and bioinformatics approaches was combined to build a focal adhesion-related signature model to predict the treatment efficacy and prognosis of patients with GC.

**Results:**

Among the various signaling pathways associated with cancer, focal adhesion was identified as a risk factor related to the treatment efficacy of chemotherapy and prognosis in patients with GC. The focal adhesion-related gene model (FAscore) discriminated patients with a high FAscore who are insensitive to neoadjuvant chemotherapy in our training cohort, and the predicted value was further verified in the GSE14210 cohort. Survival analysis also demonstrated that patients with high FAscores had a relatively shorter survival compared to those with low FAscores. In addition, we found that the levels of tumor mutation burden (TMB) and microsatellite instability (MSI) increased with an increase in FAscore, and the tumor microenvironment (TME) also shifted to a pro-tumor immune microenvironment.

**Conclusion:**

The FAscore model can be used to predict the treatment efficacy of chemotherapy and select appropriate treatment strategies for patients with GC.

## Introduction

Gastric cancer (GC) is a commonly diagnosed cancer and the leading cause of cancer-related death (1, 2). Furthermore, most of these cases are diagnosed at locally advanced stages, with high mortality (3, 4). Recent studies have found that perioperative chemotherapy, including neoadjuvant (preoperative) chemotherapy and adjuvant (postoperative) chemotherapy, can improve the survival of most patients with locally advanced GC compared with surgery combined with adjuvant chemotherapy alone (3, 5, 6). However, the therapeutic efficacy is different in patients with GC and similar clinical characteristics due to high heterogeneity (7). Our previous study (8) found that the postoperative pathology of more than half of patients who received neoadjuvant chemotherapy showed no obvious tumor regression, suggesting no benefit from the treatment. Thus, there is an urgent need to develop novel approaches that can predict the efficacy of treatment to improve the therapeutic efficacy and prognosis of patients.

Focal adhesion plays an important role in the interaction between the cell-extracellular matrix (ECM) and cell cytoskeleton and regulates cell signaling to direct cell migration, cell differentiation, proliferation, growth and response to stress (9). Furthermore, it has also been reported to be associated with various pathological processes (9), including tumor development (10). Increasing studies have shown that the activity of focal adhesion signaling can affect the response of tumor cells to treatment (11, 12). For example, focal adhesion kinase (FAK) is a key positive-related protein of focal adhesion, and upregulation of FAK expression promotes cell adhesion and metastasis (13). FAK phosphorylation can enhance focal adhesion and intrinsic resistance to cisplatin-mediated cytotoxicity in ovarian tumors (14). Melanoma cells with adaptive resistance to the targeted treatment of vemurafenib can be rendered sensitive again using FAK inhibitors (15). Mechanistic studies have revealed that these effects may be related to the regulatory function of FAK on the immune microenvironment (16). However, previous studies did not find an association between focal adhesion signatures and the treatment efficacy of neoadjuvant/adjuvant chemotherapy in patients with GC.

In the present study, we not only identified focal adhesion among the various signaling pathways as an efficacy-related factor for chemotherapy in GC but also constructed a focal adhesion-related model for predicting treatment efficacy and prognosis. Furthermore, the predictive value was confirmed in three independent cohorts. Finally, we found that the diverse treatment outcomes in the subgroups may result from alteration in the tumor immune microenvironment.

## Methods

### Patient Cohorts and Data Acquisition

A total of 729 patients with GC and follow-up information from four cohorts (n = 33, 123, 198 and 375) were included in this study. A cohort of 33 patients from our center who underwent standard perioperative chemotherapy was used as the training cohort. Of these, 15 patients responded well to preoperative chemotherapy with a necrotic area of more than 80% and 18 patients responded poorly with less than 20% necrosis. RNA sequencing was performed on the pre-treatment tumor samples. In addition, a cohort of 198 patients who received surgery and postoperative adjuvant chemotherapy and treated at the Peking University Cancer Hospital (PKUCH, Beijing, China) was also used for validation. RNA expression data GSE14210 from the National Cancer Institute of the United States of America was downloaded from the Gene Expression Omnibus (GEO) database (https://www.ncbi.nlm.nih.gov/geo/). All patients in the GSE14210 cohort were at a late stage (TNM IV stage). Twenty-two of the 123 patients who initially responded to chemotherapy developed acquired resistance. All regimens used in the three cohorts are platinum and fluorouracil combination chemotherapy (SOX: oxaliplatin + S-1; XELEX: oxaliplatin + capecitabine). The RNA-seq data and clinical parameters of patients with stomach adenocarcinoma (STAD) in the Cancer Genome Atlas (TCGA) cohort were downloaded from the UCSC website (https://xenabrowser.net/datapages/). All patients provided written informed consent, and the Ethical Committee of PKUCH approved this study.

### Pathological Evaluation of Neoadjuvant Chemotherapy

The NCCN guidelines were used to grade tumor regression following neoadjuvant chemotherapy in patients with GC (17) as follows: Grade 0, complete regression with no residual tumor cells; Grade 1, near-complete response with single cells or rare small groups of cancer cells; Grade 2, partial tumor regression, with residual cancer cells with evident tumor regression, but more than single cells or rare small groups of cancer cells; and Grade 3, extensive residual cancer with no evident tumor regression. TRG 0 was defined as a pCR. TRG 0/1 was defined as a responder to treatment, whereas TRG 3 was identified as a non-responder.

### Establishment of a Focal Adhesion-Related Model for Therapeutic Efficacy

In the training set, differentially expressed genes (DEGs) between patients with and without response to neoadjuvant chemotherapy were analyzed using the ‘limma’ R package. Gene set information for focal adhesion was obtained from the *GSEA/MsigDb* website (http://www.broadinstitute.org/gsea/msigdb/index.jsp). We then used the least absolute shrinkage and selection operator (LASSO) Cox regression model to screen for the most robust markers for treatment efficacy. We extracted the coefficients corresponding to the selected parameters in the LASSO model. Based on these results, we built a predictive model for the focal adhesion score:


$$\text{F}{\text{A}}_{\text{score}}=\sum (LASSO\;coefficient\;of\;RNAi\times RNAi\;expression)$$

The best FAscore cut-off value was determined using the X-tile software (18). The samples were divided into FAscorelow and FAscorehigh groups based on their cut-off values. We then compared the FAscores in patients with response or resistance to cisplatin and fluorouracil combination chemotherapy to validate the efficacy predictive value using the GSE14210 cohort. We also compared the differences in prognosis between the two groups to validate the prognostic value using the four included cohorts.

### Bioinformatics and Statistical Analysis

The Z-score normalization method was used to standardize the RNA expression data. The ‘limma’ R package was used to identify DEGs. Kyoto Encyclopedia of Genes and Genomes (KEGG) enrichment analysis (19) was performed using the Kobas database (http://kobas.cbi.pku.edu.cn/genelist/) (20), to identify signaling pathways related to the efficacy of neoadjuvant chemotherapy in patients with GC. R software (version 4.1.0, http://www.r-project.org), SPSS Statistics 25 (IBM Corp., Armonk, NY, USA) and GraphPad Prism 8.0 (GraphPad Software Inc., San Diego, CA, USA) were used to analyze data and plot figures. Receiver operating characteristic (ROC) analysis was performed to estimate the predictive power using the R package ‘survival’. Survival curves were plotted using the Kaplan–Meier method, and differences were evaluated using the log-rank test. A predictive model was constructed using the LASSO Cox regression model with the R package ‘LASSO’. The correlation between FAscores and TMB/MSI was analyzed using Home-for-researchers website (www.home-for-researchers.com/static/index.html#/). Chi-square tests were performed to analyze the categorical variables. Cibersortx software (https://cibersortx.stanford.edu) was used to identify the cell-type-specific gene expression signatures (21) and immune cell subtypes were analyzed using the cibersort method. All statistical tests were two-sided, and statistical significance was ​​set at *p* < 0.05.

## Results

### Focal Adhesion was Identified as a Relevant Factor for the Treatment Efficacy of Neoadjuvant Chemotherapy in Patients with GC

A total of 33 patients with GC (clinical stage II-III) who received neoadjuvant chemotherapy and D2 gastrectomy plus lymphadenectomy were included in the training set. After neoadjuvant chemotherapy, treatment efficacy was evaluated by estimating the extent of tumor regression on postoperative pathology according to the NCCN guidelines (17). Fifteen patients responded well (TRG 1) to preoperative chemotherapy with a tumor necrosis rate of more than 80%, and 18 patients responded poorly (TRG 3) with less than 20% necrosis. The baseline clinicopathological data of the 15 responders and 18 non-responders were analyzed and the results showed that non-responders had a higher ratio of lymph node metastasis (*p* = 0.039, **Table 1**) and later cTNM stage (*p* = 0.016, **Table 1**).

**Table 1:** Analyses of relative factors for treatment response to chemotherapy in patients with gastric cancer.

Characteristics	Responder (n = 15)	Non-responder (n = 18)	*P* value
Gender			0.182
Female	2 (13.3%)	6 (33.3%)	
Male	13 (86.7%)	12 (66.7%)	
Age			0.056
<59 (median)	10 (66.7%)	6 (33.3%)	
>59 (median)	5 (33.3%)	12 (66.6%)	
Tumor volume (cm^3^)			0.849
<6.9 (median)	7 (46.7%)	9 (50.0%)	
>6.9 (median)	8 (53.3%)	9 (50.0%)	
cT			0.741
III	5 (33.3%)	7 (38.9%)	
IV	10 (66.7%)	11 (61.1%)	
cN			0.039*
0	5 (33.3%)	1 (5.6%)	
I-III	10 (66.7%)	17 (94.4%)	
cM			0.999
0	15 (100%)	18 (100%)	
cTNM			0.016*
II	6 (40.0%)	1 (5.6%)	
III	9 (60.0%)	17 (94.4%)	
FAscore			<0.001***
Low	15 (100%)	3 (16.7%)	
High	0 (0.0%)	15 (83.3%)	

*p < 0.05, ***p < 0.001.

**Figure 1:**
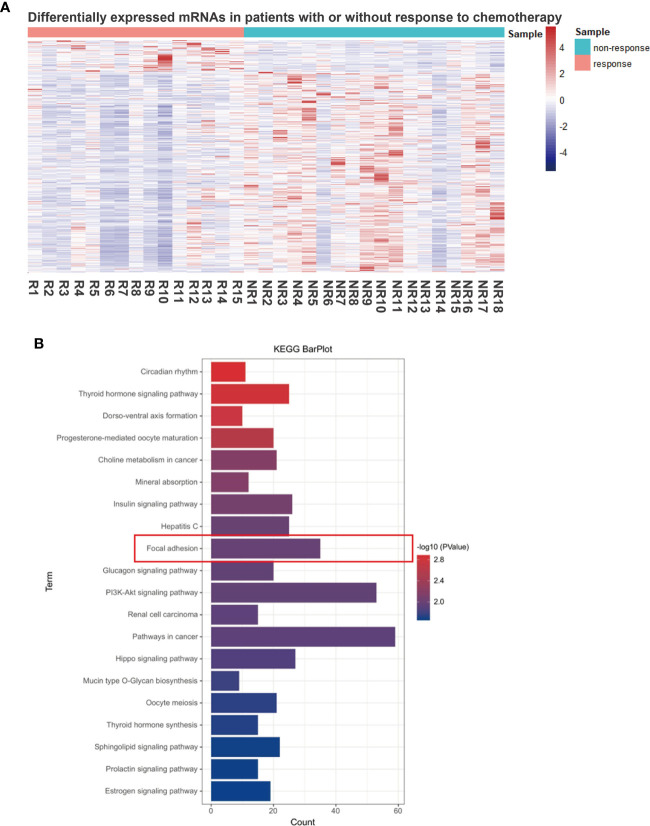
Focal adhesion is a significantly relevant pathway for the treatment efficacy of neoadjuvant chemotherapy in GC. **(A)** Differentially expressed genes (DEGs) between the responders (tumor regression grading 1, TRG 1) and non-responders (TRG 3). **(B)** The functional enrichment of DEGs was assessed based on Kyoto Encyclopedia of Genes and Genomes (KEGG) pathways and focal adhesion was identified.

**Figure 2:**
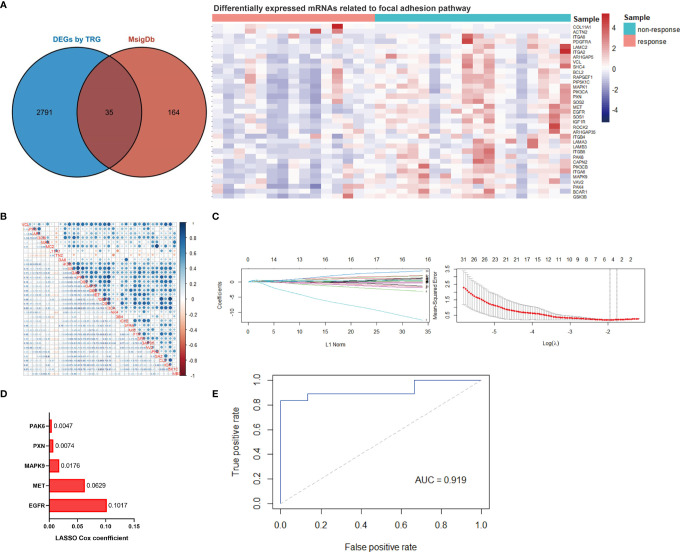
Establishment of the focal adhesion-related gene signature for predicting efficact to chemotherapy in patients with gastric cancer. **(A)** Thirty-five focal adhesion-related genes were identified among the DEGs between responders and non-responders to chemotherapy. **(B)** The linear correlation analysis of 35 identified focal adhesion-related genes. **(C)** The most robust predictive genes were identified using the least absolute shrinkage and selection operator (LASSO) Cox regression algorithm. **(D)** An ensemble of 5 genes remained with nonzero coefficients. **(E)** Receiver operating characteristic (ROC) analysis for the predictive power of the focal adhesion-related gene signature, and the area under the ROC curve was 0.919.

We then performed RNA sequencing of these 33 gastric biopsy samples during gastroscopy prior to neoadjuvant chemotherapy and 33,828 genes were identified. Subsequently, 2,830 DEGs between responders and non-responders were identified using an R package ‘limma’ (**Figure 1A**). To evaluate relevant signaling pathways of efficacy, the KEGG pathway analysis was performed based on the Kobas database (http://kobas.cbi.pku.edu.cn/genelist/) (20). Among the various signaling pathways associated with cancer, focal adhesion was identified as a risk factor related to the treatment efficacy of neoadjuvant chemotherapy (*p* < 0.0001, **Figure 1B**). Subsequently, we obtained the gene sets of focal adhesions from the *GSEA/MsigDb* website (http://www.broadinstitute.org/gsea/msigdb/index.jsp), and 199 genes were identified (**Supplementary Table 1**). Furthermore, 35 of these patients (17.6%) were found to be significantly associated with response to neoadjuvant chemotherapy in GC (**Figure 2A**). These results suggest that focal adhesions are an indicator of the treatment efficacy of neoadjuvant chemotherapy in GC.

### Establishment and Validation of a Focal Adhesion-Related Model for Therapeutic Efficacy

Collinearity among focal adhesion DEGs was detected and identified (**Figure 2B**). The LASSO Cox regression model was then used to screen the most robust markers for treatment efficacy. Ten-fold cross-validation was used to overcome over-fitting, and an optimal λ value of 0.1423 was selected (**Figure 2C**). An ensemble of five genes (EGFR, MET, MAPK9, PXN and PAK6) remained with individual nonzero coefficients (**Figure 2D**), which were integrated to establish a focal adhesion-related predictive signature (FAscore) with an intercept of 0.545454. The network of potential interactions between focal adhesion-related DEGs was analyzed, and it was observed that three genes (EGFR, MET and MAPK9) from our established model were strongly associated with other genes (**Supplementary Figure 1**), demonstrating a good representation of the focal adhesion pathway.

**Figure 3:**
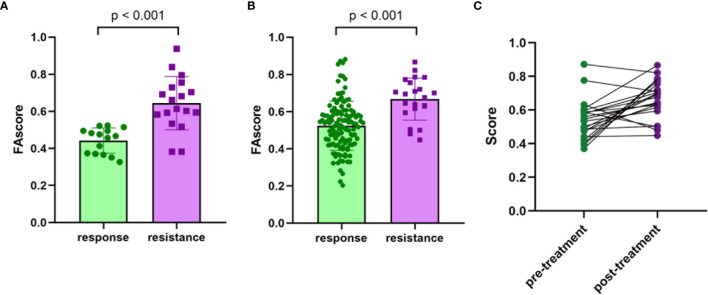
Validation of the efficacy predive value. **(A)** The values of the focal adhesion-related model score (FAscore) in patients with response and resistance to neoadjuvant chemotherapy in the training set. **(B)** The FAscores in the response and acquired resistance groups to cisplatin and fluorouracil combination chemotherapy in the GSE14210 cohort. **(C)** The FAscores of samples before and after developing resistance to chemotherapy in the GSE14210 cohort.

Then, ROC analysis was performed based on calculated FAscores, and the result showed that the area under the ROC curve (AUC) was 0.919 (**Figure 2E**). These results showed that this FAscore model was an independent factor for treatment response to chemotherapy in GC and could robustly discriminate patients with different treatment efficacies. To further validate the predictive power, we compared the FAscores in the responders and non-responders from the training set and estimated the differences using the Wilcoxon test. A significantly higher FAscore was observed in patients who responded poorly to treatment (*p* < 0.001, **Figure 3A**). We also compared the FAscores in paired response-acquired resistance samples from patients treated with cisplatin and fluorouracil combination chemotherapy from the GSE14210 cohort. Before cisplatin and fluorouracil treatment, endoscopic biopsy samples from 123 patients with late-stage GC (TNM IV stage) were collected. Twenty-two patients were re-biopsied after developing resistance to treatment. Then, RNA sequencing was performed on the 123 initial samples and 22 resistant specimens, and the FAscores were calculated. The results showed that 22 resistant specimens had a higher FAscore (*p* < 0.001, **Figure 3B**). Furthermore, the FAscore alterations associated with acquired chemotherapy resistance in pre-and post-biopsy samples from the same patient were also analyzed. Among the 22 patients who developed acquired chemotherapy resistance, 18 (81.8%) had an increased FAscore (**Figure 3C**).

### Prognostic Value of the Established FAscore

**Figure 4:**
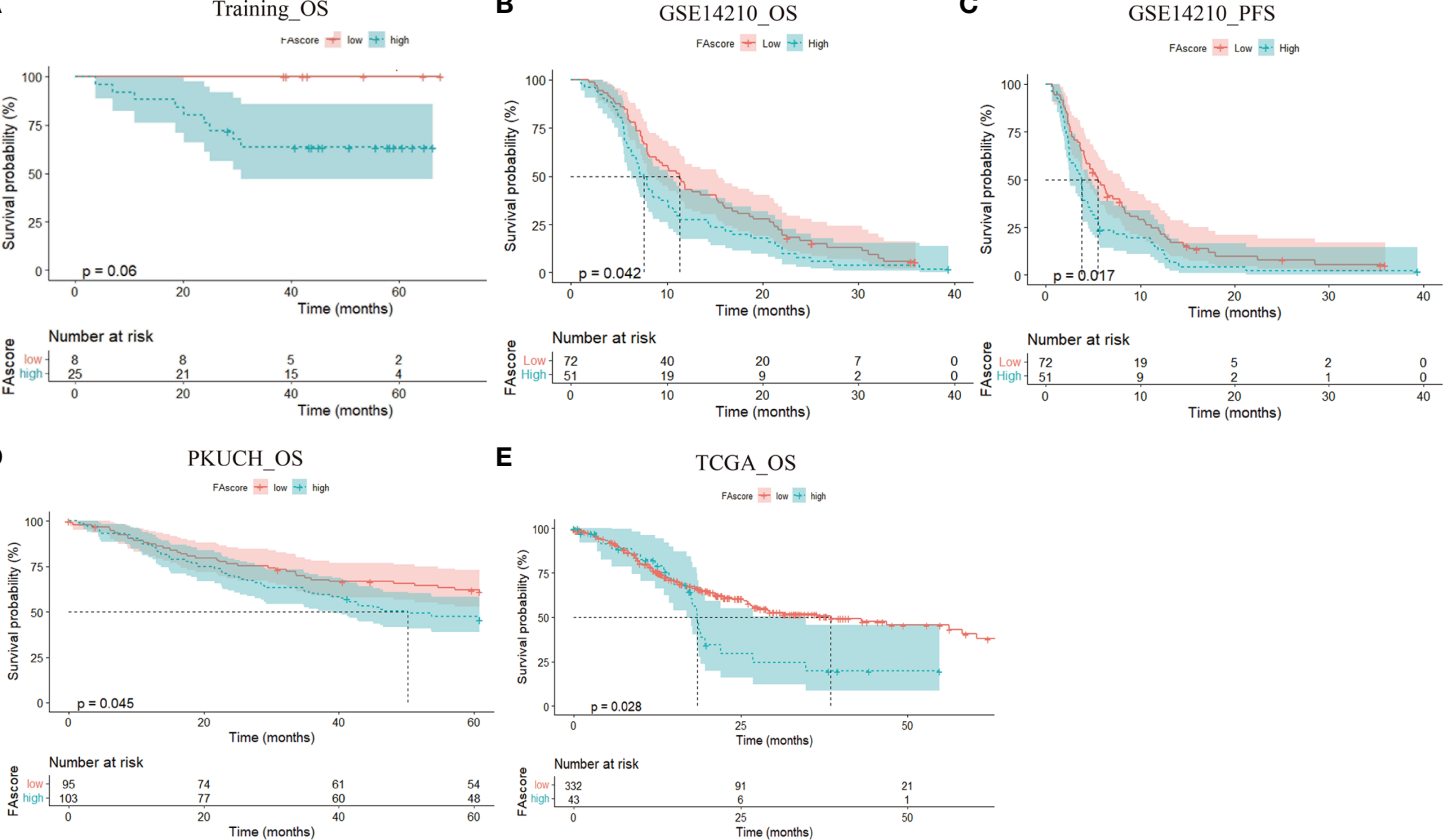
The prognostic value of the established focal adhesion-related model. High FAscore discriminated poor prognosis patients in different cohorts, including the training cohort **(A)**,GSE14210 cohort **(B, C)**, Peking University Cancer Hospital (PKUCH) cohort **(D)**, and the Cancer Genome Atlas (TCGA) cohort **(E)**.

Generally, a good response to treatment is associated with a favorable survival. We investigated whether the FAscore can discriminate between good and poor prognosis patients among the training cohort, PKUCH cohort, GSE14210 cohort and TCGA cohort. In the training cohort, we found that patients with high FAscores had a shorter survival time than those with low FAscores (**Figure 4A**) with a cut-off value of 0.40, which was determined using the X tile software (18). This is consistent with the situation of efficacy prediction, and is expected. In agreement with the above data, the survival analyses in validation groups verified that patients with high FAscores had shorter survival times (**Figures 4B–E**). Thus, the focal adhesion-related model also had a good predictive effect on prognosis.

### Relationship Between the Focal Adhesion-Related Signature and Tumor Mutation Burden/Microsatellite Instability

**Figure 5:**
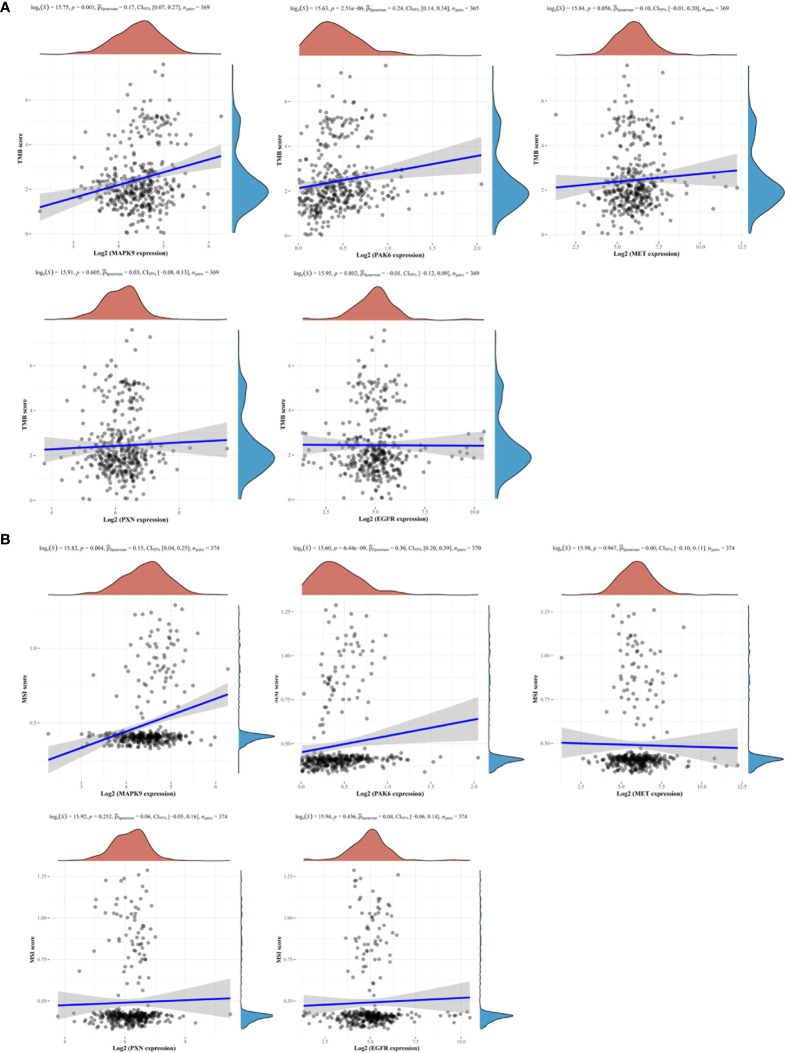
Focal adhesion-related gene signatures and tumor mutation burden (TMB)/microsatellite instability (MSI). Two focal adhesion-related gene signatures (MAPK9, PAK6) were observed to be positively associated with the levels of TMB **(A)** and MSI **(B)**.

Our previous studies have identified that patients with high-level TMB (TMB-H) or high-level MSI (MSI-H) had a worse treatment benefit from neoadjuvant chemotherapy in GC (8, 22). We then analyzed the association between the model genes and TMB/MSI based on the TCGA cohort. The results indicated that the expressions of MAPK9, PAK6 and MET were markedly correlated with a higher TMB (**Figure 5A**). Consistent with the TMB alterations, MSI-H was also positively related to MAPK9 and PAK6 expression (**Figure 5B**). These results demonstrated that increased FAscores indicated higher levels of TMB and MSI.

### Focal Adhesion-Related Model Prompted an Immune Microenvironment Change

**Figure 6:**
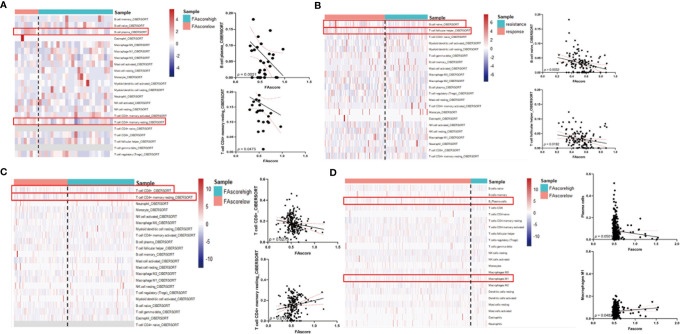
High FAscores indicated a pro-tumor immune microenvironment. Analysis of the correlation between FAscores and the proportions of immune cell subpopulations in different cohorts, including the training cohort **(A)**, GSE14210 cohort **(B)**, Peking University Cancer Hospital (PKUCH) cohort **(C)**, and the Cancer Genome Atlas (TCGA) cohort **(D)**. The results showed that high FAscores indicated an decrease in the ratio of anti-tumor immune cells.

**Figure 7:**
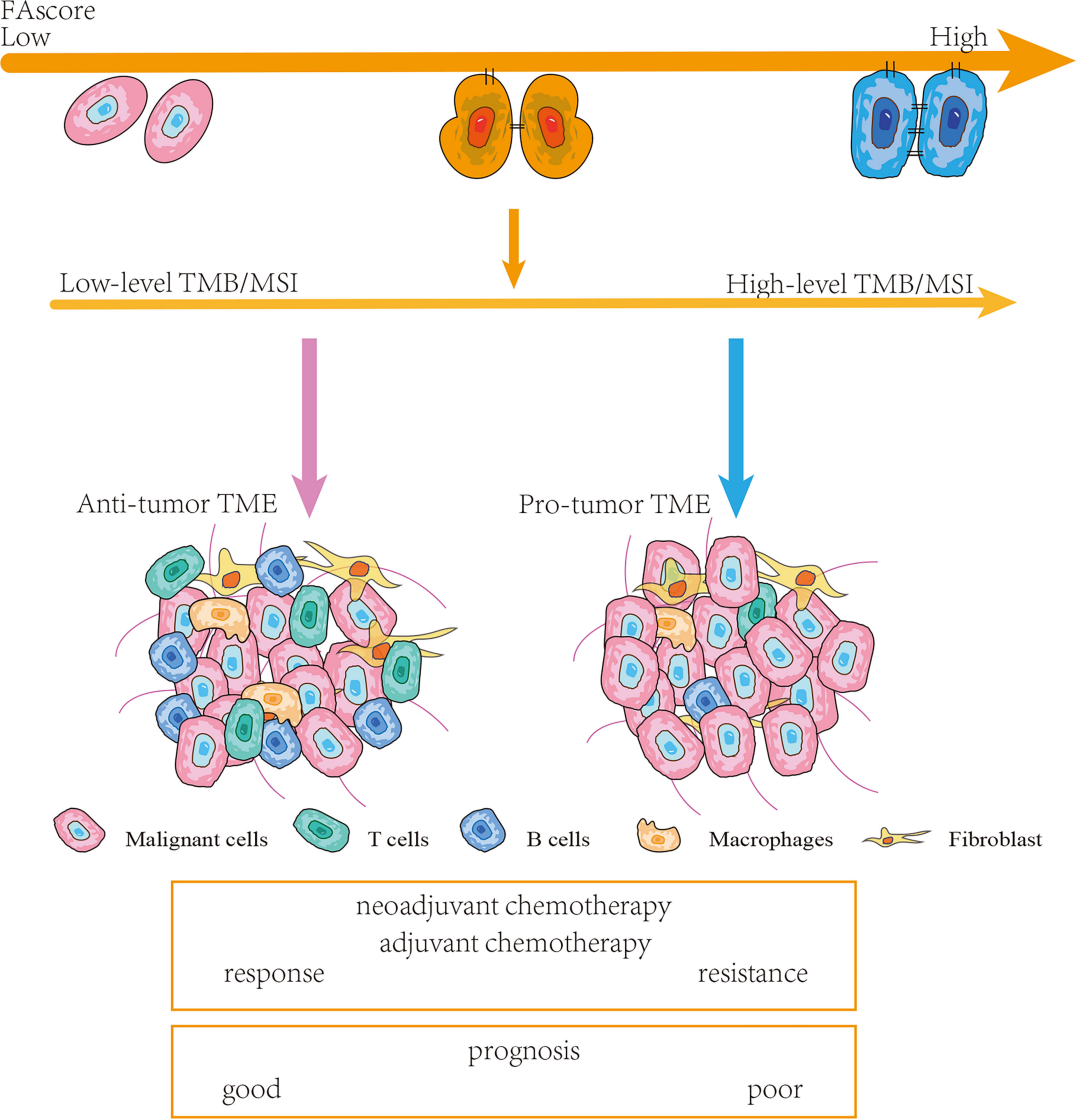
Schematic diagram. Patients with high FAscores were frequently accompanied by high levels of tumor mutation burden (TMB)/microsatellite instability (MSI) and pro-tumor tumor microenvironments (TMEs). Thereby, such patients were more likely to respond poor to chemotherapy and had a shorter survival time.

The tumor immune microenvironment plays a vital role in cancer progression and is associated with the treatment efficacy of chemotherapy (23). We investigated the proportions of distinct immune cell subpopulations in tumor samples using the CIBERSORT algorithm (24). Relative fractions of 22 immune cells in each tumor specimen were identified and the association between immune cell proportions and FAscores was estimated using linear regression. Six immune cell subtypes were identified as factors affecting the focal adhesion-related model, including plasma B cells, naïve B cells, resting memory CD4+ T cells, follicular helper T cells, CD8+ T cells and M1 polarized macrophages (**Figures 6A–D**). Conclusively, several anti-tumor immune cells, including plasma B cells, naïve B cells, follicular helper T cells and CD8+ T cells, significantly decreased (*p* < 0.01) with an increase in FAscore. In terms of tumor correlation, the tumor immune microenvironment changed from an anti-tumor phenotype to a pro-tumor phenotype as the FAscore increased (**Figure 7**), resulting in a poor response to neoadjuvant/adjuvant chemotherapy in patients with GC.

## Discussion

Radical gastrectomy plus postoperative adjuvant chemotherapy has been the standard treatment of patients with advanced GC for the last few decades (25, 26). However, patients with advanced GC frequently harbor micrometastases, which are rarely detected by preoperative examination and result in poor prognosis (27). Therefore, neoadjuvant chemotherapy has been considered and developed. NACT has several potential advantages, including tumor down-staging, increasing the rate of curative resection, clearing micrometastases, and decreasing locoregional recurrence (25, 28). Furthermore, post-treatment tumor tissues are available after gastrectomy, and therapeutic efficacy can be directly evaluated by pathology. We then performed RNA sequencing on pre-treatment samples from 33 patients who received neoadjuvant chemotherapy and evaluated the treatment efficacy by postoperative pathology according to the NCCN guidelines. This cohort was also used as the training set to accurately screen for signals related to treatment efficacy.

Among the various signaling pathways of cancer, we identified focal adhesion as a significant pathway for the therapeutic efficacy of neoadjuvant chemotherapy using KEGG enrichment (19). The focal adhesion-related DEGs between responders and non-responders were identified using the ‘limma’ R package and the *GSEA/MsigDb* website. Subsequently, the LASSO Cox regression model was used to screen robust predictive biomarkers for treatment efficacy in order to construct a focal adhesion-related gene signature. Furthermore, the predictive capacity was verified in the GSE14210 cohort. Notably, in four independent cohorts (the training cohort, PKUCH cohort, UCIUSA cohort, and TCGA cohort), the focal adhesion-related gene signature also had a promising prognostic value.

According to the current understanding of the GC genome, no hotspot mutations or specific gene deletions/amplification have been identified (25). However, previous studies found that TMB, a genetic alteration at the whole genome level, is associated with tumor progression and the prognosis of patients with GC (29). It is well known that somatic non-synonymous variants (SNVs) can enhance tumor immunogenicity by expressing neoantigens (30) and further cause more tumor immune infiltration, which is associated with the prognosis of patients (31). Furthermore, TMB shows a negative relationship with the treatment efficacy of chemotherapy in GC (22, 32). In addition to TMB, MSI is also an indicator of the prognosis and treatment response, especially in immunotherapy (33, 34). Our previous study also showed that patients with MSI-H responded poorly to preoperative chemotherapy (8). In this study, we analyzed the association between focal adhesion-related gene signatures and TMB/MSI. The results showed that the expression levels of MAPK9, PAK6, and MET were positively correlated with TMB-H, while MAPK9 and PAK6 were positively associated with MSI-H.

Recently, the development of single-cell sequencing technology has enabled the analysis of tumor microenvironmental components at a higher resolution (35, 36). A variety of cell subpopulations have been identified in GC, and these subpopulations exhibit completely different biological and immunological functions, thus revealing the high complexity of the microenvironment (37, 38). In addition, studies on a variety of tumors have also suggested that different types of cancer may have similar immune cell types, but their proportions are different (36). This difference inevitably affects the occurrence and development of tumors. Furthermore, it has implications in predicting the prognosis and treatment sensitivity of patients. In the current study, the proportion of immune cells was analyzed using the Cibersort algorithm. Our focal-adhesion model based on these immune microenvironmental components also showed a good predictive ability. The proportion of some anti-tumor immune cells, including CD8+ T cells, plasma B cells and follicular helper T cells, decreased with an increase in FAscore. These results suggested that the immune environment transformed into pro-tumor roles with an increasing FAscore.

Although a promising predictive capacity of the focal adhesion-related mode was observed both in the training and validation sets, several issues need to be explored and elucidated in the future: First, this study is a retrospective study, and the predictive value of our focal adhesion-related signatures needs further verification in larger prospective studies. Second, further experimental studies are required to elucidate the focal adhesion-related biological functions underlying the gene signature in GC.

## Conclusion

In summary, we constructed a novel focal adhesion-related model (FAscore) to predict the treatment efficacy of chemotherapy in patients with GC. The model can screen patients who are sensitive to treatment and thus facilitate personalized GC management. Furthermore, this predictive model also had a prognostic value, and patients with a low FAscore were predicted to have a favorable survival. In addition, the increase in FAscore suggested a higher level of TMB and MSI, and a pro-tumor phenotype of the TME.

## Data Availability Statement

Publicly available datasets were analyzed in this study. This data can be found here: GSE14210 at https://www.ncbi.nlm.nih.gov/geo/ and TCGA stomach cancer (STAD) at https://xenabrowser.net/datapages/. The original data presented in this study are included in the article or supplement materials, further inquires can be directed to the corresponding authors.

## Ethics Statement

All patients provided written informed consent, and the Ethical Committee of PKUCH approved this study.

## Author Contributions

XT, XW and TG contributed equally to this work. XG and ZL had full access to all of the data in the study and take responsibility for the integrity of the data and the accuracy of the data analysis. Concept and design: XT, XG and ZL. Acquisition, analysis, or interpretation of data: All authors. Drafting of the manuscript: XT, XW and TG. Critical revision of the manuscript for important intellectual content: TG, XG and ZL. Statistical analysis: XT and XW. Obtained funding: XG and ZL. Administrative, technical, or material support: XW, TG, FJ, XX and YH. Supervision: YH, XG and ZL. All authors contributed to the article and approved the submitted version.

## Funding

This study was funded by National Natural Foundation of China (31870805), Hospital Management Center in Beijing (QML20201104), Beijing Cancer Hospital (A002431) and Chinese Society of Clinical Oncology Research Foundation (Y-HR2019-0375).

## Conflict of Interest

The authors declare that the research was conducted in the absence of any commercial or financial relationships that could be construed as a potential conflict of interest.

## Publisher’s Note

All claims expressed in this article are solely those of the authors and do not necessarily represent those of their affiliated organizations, or those of the publisher, the editors and the reviewers. Any product that may be evaluated in this article, or claim that may be made by its manufacturer, is not guaranteed or endorsed by the publisher.

## Supplementary Material

Supplementary Figure 1 The network of potential interactions among focal adhesion-related differentially expressed genes (DEGs) to treatment response.
